# Using CRISPR-Cas9 to quantify the contributions of O-glycans, N-glycans and Glycosphingolipids to human leukocyte-endothelium adhesion

**DOI:** 10.1038/srep30392

**Published:** 2016-07-26

**Authors:** Gino Stolfa, Nandini Mondal, Yuqi Zhu, Xinheng Yu, Alexander Buffone, Sriram Neelamegham

**Affiliations:** 1 Department of Chemical & Biological Engineering and Clinical & Translational Research Center, State University of New York, Buffalo, NY 14260, USA

## Abstract

There is often interest in dissecting the relative contributions of the N-glycans, O-glycans and glycosphingolipids (GSLs) in regulating complex biological traits like cell signaling, adhesion, development and metastasis. To address this, we developed a CRISPR-Cas9 toolkit to selectively truncate each of these commonly expressed glycan-types. Here, O-glycan biosynthesis was truncated by knocking-out Core 1 β3Gal-T Specific Molecular Chaperone (*COSMC*), N-glycans by targeting the β1,2 GlcNAc-transferase (*MGAT1*) and GSLs by deleting UDP-glucose ceramide glucosyltransferase (*UGCG*). These reagents were applied to reveal the glycoconjugates regulating human myeloid cell adhesion to selectins under physiological shear-flow observed during inflammation. These functional studies show that leukocyte rolling on P- and L-selectin is ablated in cells lacking O-glycans, with N-glycan truncation also increasing cell rolling velocity on L-selectin. All three glycan families contributed to E-selectin dependent cell adhesion with N-glycans contributing to all aspects of the leukocyte adhesion cascade, O-glycans only being important during initial recruitment, and GSLs stabilizing slow cell rolling and the transition to firm arrest. Overall, the genome editing tools developed here may be broadly applied in studies of cellular glycosylation.

N-linked glycans, O-glycans and glycosphingolipids (GSLs) represent three common forms of glycoconjugates on mammalian cell surfaces. Their interaction with carbohydrate binding proteins or lectins control a number of cellular processes including cell signaling, inflammation and cancer metastasis^1^,^2^,^3^,^4^. While the core structures that initiate glycan biosynthesis on these different glycoconjugates is distinct, chain extension is often similar, with lactosamine repeats that are capped by sialic acid and/or fucose^5^. Due to this, terminal epitopes like the sialyl Lewis-X (sLe^X^) (NeuAcα2,3Galβ1,4[Fucα1,3]GlcNAc) or blood group antigens that represent the functional end group are often common between the N-, O- and lipid- linked glycans. Due to this structural overlap, it is often important to identify the unique underlying glycoconjugate scaffolds that express these functional end groups.

Current methodologies to decipher the relative contributions of N- and O-linked glycoproteins and GSLs to cell function include the use of small molecule inhibitors that block the biosynthesis of O-glycans (e.g. benzyl-α-GalNAc)^6^,^7^ or glycosphingolipids (e.g. 1-phenyl-2-palmitoylamino-3-pyrrolidino-1-propanol derivatives)^8^, enzymatic cleavage of N-glycans (PNGase-F)^9^ or gene silencing of glycosyltransferase expression with shRNA^10^,^11^. Though these tools provide useful information, they are each limited by the incomplete elimination of the glycans of interest and possible non-specific effects, making unambiguous interpretation of data difficult. Genome editing can overcome these limitations, with CRISPR (clustered regularly interspaced short palindromic repeats)-Cas9 being the simplest and most rapid technology^12^. Here, targeted, efficient double strand breaks (DSBs) catalyzed by the Cas9 nuclease is repaired by error-prone non-homologous end-joining (NHEJ)^12^. This results in base insertions or deletions (indels), leading to frameshift mutations and truncated protein expression.

The current manuscript describes the use of the CRISPR-Cas9 method to dissect, for the first time, the relative contributions of O-glycans, N-glycans and GSLs during shear-dependent selectin mediated human cell adhesion. We study this molecular interaction due to the broad importance of carbohydrate ligands that function as selectin-ligands during hematopoietic stem and progenitor cell (HSPC) homing to the bone marrow vascular niche^13^, cancer cell metastasis^3^, and leukocyte trafficking during immunity and inflammation^4^,^14^. Here, the relative roles of O-glycans, N-glycans and GSLs to molecular binding can vary in a cell-type and context-specific manner^15^,^16^,^17^. For example, O-linked glycans expressed on peripheral node addressins (PNAd) were considered to exclusively facilitate L-selectin mediated lymphocyte homing, until Mitoma *et al*.^15^ demonstrated a critical role for N-glycans as well using mice lacking O-glycan biosynthesis pathways. While the N-glycans on CD44 facilitate HSPC binding to E-selectin, it is the CD44 O-glycans that facilitate E-selectin recognition in LS174T colon carcinoma cells^17^. Finally, the E-selectin ligands in human neutrophils are different from that in mice since E-selectin exclusively binds glycoproteins in mice, but it also engages GSLs in humans^16^,^18^. Overall, easy-to-use tools to quantify the roles of the different glycoconjugates are needed for diverse studies related to cell adhesion, migration and signaling.

As a starting point, with the goal of quantifying the glycoconjugates responsible for selectin engagement in humans during inflammation, the current study generated a panel of mutant promyeloid leukemic HL-60 cells. We choose to particularly focus on human cells since recent studies show that the glycans participating in human leukocyte adhesion are likely different from other mammals, most notably mice^10^,^11^,^16^,^18^,^19^. Yet knowledge regarding the human selectin-ligands is incomplete, especially as it relates to the E-selectin ligands^14^,^19^. Further, we select HL-60 s since these cells bear close resemblance to human neutrophils in terms of glycosyltransferase expression as well as selectin binding phenotype^20^. Such studies are currently not possible in short-lived primary *human* neutrophils since CRISPR-Cas and related technologies still need to be developed to efficiently knock-out multiple genes in the hard-to-transfect primary cells, and specifically track these knockouts in functional assays.

The specific enzyme/enzyme-chaperones disrupted include (Fig. 1): i. Core-1 specific molecular chaperone COSMC (*C1GalT1C1*) to target O-glycans since this protein is necessary for Core-1 Galactosyltransferase T-synthase (*C1GalT1*) activity and O-glycan extension^21^ (Fig. 1a). ii. UDP-Glucose Ceramide Glucosyltransferase (*UGCG*) to ablate glucosylceramide biosynthesis since this enzyme exclusively adds the first glucose to ceramide substrates^22^ (Fig. 1b). iii. Mannosyl (α1,3)-glycoprotein β1,2-N-acetylglucosaminyltransferase MGAT1 (*GlcNAcT1*) to truncate N-glycans as this enzyme is necessary for hybrid and complex N-glycan biosynthesis^23^ (Fig. 1c). Since the ER mediated steps in N-glycan formation are not altered, this mutation is not expected to affect protein folding^24^. Cells lacking O-glycans, GSLs and N-glycans generated in this manner are abbreviated [O]^−^, [G]^−^ and [N]^−^ respectively. In addition, dual knockouts (KOs) that either only express O-glycans ([NG]^−^ cells), GSLs ([NO]^−^) or N-glycans ([OG]^−^) and triple KOs ([NOG]^−^, [GON]^−^) were also generated. Thus, in addition to ‘lack-of-structure’ experiments, complementary ‘retention-of-structure’ assays were also possible to confirm specific findings. Detailed cell adhesion studies performed under shear confirm the primary role of core-1 derived O-glycans in mediating cellular interactions with P- and L-selectin on HL-60s^25^, with a smaller role for N-glycans in regulating L-selectin dependent cell rolling. In the case of human E-selectin ligands, N-linked glycans contributed to all aspects of the leukocyte adhesion cascade, with O-glycans being important during initial recruitment, and GSLs stabilizing slow cell rolling and the transition to firm arrest. Overall, the manuscript provides a toolkit to quantify the relative contributions of N-, O- and lipid-linked glycoconjugates to myeloid cell selectin-mediated interactions, which can be applied to study other cellular processes also.

## Results

### HL-60 knockouts lacking one or more glycosyltransferases

**Figure 1:**
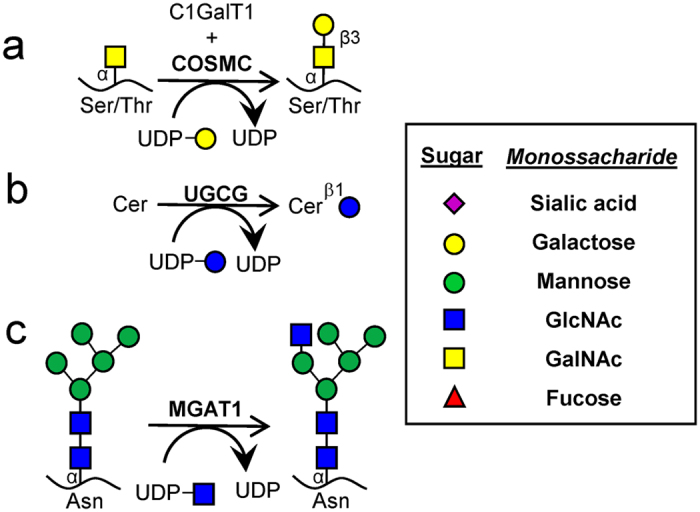
Schematic for the initiation of O-linked, lipid linked and N-linked glycosylation. (**a**) Core-1 derived O-glycan biosynthesis begins with the formation of the T-antigen using the T-synthase C1GalT1 and its chaperone COSMC. (**b**) UGCG adds the first Glc to ceramide to form Glc-Cer glycolipids. (**c**) MGAT1 catalyzes the addition of GlcNAc to the Man-5 substrate. Knocking out these three key enzymes results in premature termination of specific types of glycoconjugates. All figures use the Consortium for Functional Glycomics symbolic nomenclature (shown in legends).

CRISPR-Cas9 was used to disrupt enzymatic reactions required for the extension of O-glycans by deleting *COSMC*, GSLs by targeting *UGCG*, and N-glycans by knocking-out *MGAT1* (Fig. 1).

**Figure 2:**
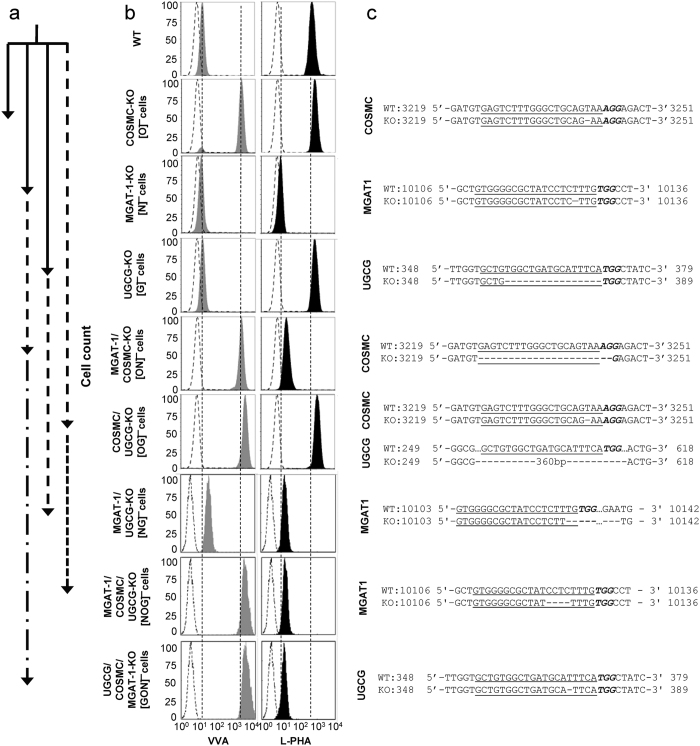
Generation of glycosyltransferase KO clones. (**a**) Arrows represent the workflow for creating KOs. Arrows lead from original cell to KO. Some double and triple -KOs were generated by serial genome editing. Thus, genome edits made in one step remain in all subsequent editing steps. Solid and dashed lines lead to single (―), double (− −) or triple-KOs (― • ―). There is only one copy of the COSMC, MGAT1 and UGCG genes in HL-60s. (**b**) Flow cytometry measurement of VVA and L-PHA lectin binding to glycoT-KO clones. Knocking out O-glycans by targeting COSMC augments VVA-lectin binding. Knocking out MGAT1 reduces L-PHA binding. Additional lectin staining data are presented in the Supplemental Material section. (**c**) Sanger sequencing results for individual KO-clones is shown next to corresponding cytometry plots. In each case, wild-type sequence is shown on the first line with target gRNA underlined and PAM/NGG sequence in bold-italicized fonts. Lower line shows sequencing results for the individual KOs with dashes representing gene deletions. For simplicity, cells having truncated O-glycans, N-glycans and glycolipids are abbreviated [O]¯, [N]¯, [G]¯ respectively (see panel b). Double and triple KOs are also similarly defined.

Combinatorial gene deletion resulted in eight different HL-60 cell lines that were deficient in one or more of the above enzymes (Fig. 2). Here, the single knockout [O]^−^, [N]^−^ and [G]^−^ cell lines, and dual knockout [OG]^−^ were created in one-step by electroporating the pertinent CRISPR vectors into WT HL-60 cells (Fig. 2a). The binding of VVA-lectin, which recognizes the Tn-antigen, was increased in the [O]^−^ knockout (Fig. 2b). The [N]^−^ cells did not bind L-PHA, a lectin recognizing hybrid and complex N-glycans. These two cell lines were FACS sorted based on lectin binding to obtain isogenic single-cell clones. Isogenic [G]^−^ and [OG]^−^ clones were similarly obtained, only single-cell sorting was performed without any fluorescent marker. Double ([NG]^−^, [ON]^−^) and triple KO (TKO) ([NOG]^−^, [GON]^−^) cells were also made by electroporating previously verified single and dual KO clones (Fig. 2a).

All clones with *COSMC* mutations had ~100-fold higher VVA binding than WT (Fig. 2b, left column). *MGAT1* deletion resulted in almost complete loss of L-PHA binding (Fig. 2b, right column). There was no change in VVA or L-PHA binding in cells that were not specifically targeted for the loss of O- or N-glycan biosynthesis. In additional studies (Supplemental Fig. S1): i. PNA binding was diminished in cells lacking O-glycans consistent with the notion that this lectin recognizes Galβ1,3GalNAc on core-1 structures; ii. ConA binding to high mannose glycans was augmented in MGAT1 knockouts as anticipated though the signal-shift was not as pronounced as that of L-PHA; iii. The Mal-II staining pattern suggests that α(2,3)sialylation predominantly occurs on O-glycans in HL-60s; and iv. ECL lectin staining of unsialylated lactosamine was reduced in cells lacking extended N-glycans and also GSLs. Thus, lactosamine chains are primarily associated with such glycoconjugates in HL-60. PNA, Mal-II and ECL staining was low in the [NOG]^−^ TKOs. The presence of desired mutations at the target site was confirmed in each case by Sanger sequencing (Fig. 2c). Off-target editing was absent based on sequencing of various computationally predicted exonic off-target genes (Supplemental Tables S2 and S3).

**Figure 3:**
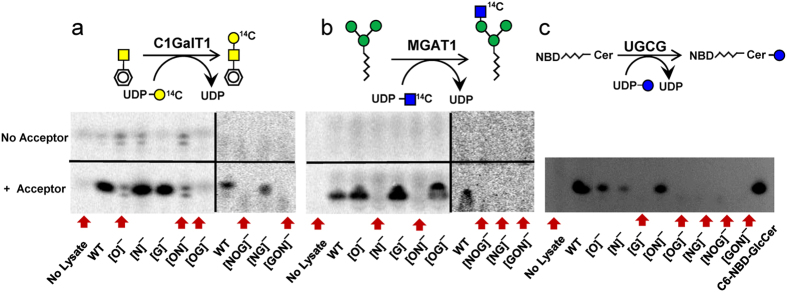
Enzymatic activity of glycoT KO clones. C1GalT1 (panel a), MGAT1 (panel b) and UGCG (panel c) enzyme activity was measured in WT and KO cell lines using the following acceptors: GalNAc-O-Benzyl for C1GalT1, man3-octyl for MGAT1 and fluorescent C6-NBD-ceramide for UGCG. Schematic representations of the individual reactions is presented above each TLC plate image captured using either phosphorimaging (**a**,**b**) or fluorography (**c**). In (**a**,**b**), the upper portion of the image presents independent control runs performed for each cell line in the absence of the acceptor substrate. This is not necessary in panel c, due to the use of a fluorescent substrate. Some lanes in panel a have minor background in both acceptor and ‘no acceptor’ controls. Knocking out specific genes abrogates corresponding enzyme activity, shown using red arrows to indicate missing reaction products.

Enzymology confirmed the deletion of specific glycosyltransferase activities in the clones (Fig. 3). Here, compared to WT HL60s, cells with *COSMC* deletion lacked O-glycan forming C1GalT1 activity that enables Gal addition to the benzyl-α-GalNAc substrate (Fig. 3a). *MGAT1* deletion similarly abolished [^14^C]GlcNAc transfer to Mannose-3-octyl (Fig. 3b). Cells with *UGCG* deletion did not form the C6-NBD-GlcCer product (Fig. 3c).

Overall the lectin staining, genome sequencing and enzymology data demonstrate the creation of a panel of isogenic human leukocyte variants. While functional results are presented below for the single clones from Fig. 2, similar data were obtained using additional clones and also sorted mutant cells containing a mixed population. Additionally, there was no obvious effect of the gene deletion sequence in that both the [NOG]^−^ and [GON]^−^ cells were comparable in all the assays. None of the cells displayed overt unusual cellular morphology or reduced proliferation based on microscopy examination (Supplemental Fig. S2).

### Changes in cell surface carbohydrate epitopes accompany genome editing

Flow cytometry evaluated changes in the expression of previously identified putative selectin-binding ligands and related carbohydrate epitopes in WT and knockout cells.

In the ligand expression studies (Supplemental Fig. S3), L-selectin expression was negligible in HL-60. Mac-1, CD43 and CD44 were similar in all cell lines, though the sialic-acid dependent anti-CD43 mAb MEM59 displayed reduced binding to clones lacking elaborated O-glycans (i.e. [O]^−^, [ON]^−^, [OG]^−^ and [NOG]^−^ cells). This is consistent with the notion that a majority of the CD43 sialoglycans are O-linked. PSGL-1 levels monitored by mAb KPL-1 (binds N-terminus of PSGL-1) was also decreased in some of the knockouts, particularly those lacking O-glycans.

**Figure 4:**
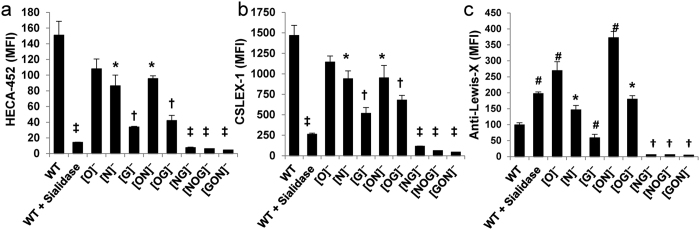
Carbohydrate epitope expression on KO cell lines. Flow cytometry measurements for the expression of the: (**a**) Cutaneous Lymphocyte Antigen (CLA) defined by mAb HECA-452, (**b**) CD15s/sLe^X^/sialyl Lewis-X using mAb CSLEX-1 and (**c**) Lewis-X/CD15 using mAb HI98. MFI: mean fluorescence intensity. * *P* < 0.05 with respect to WT HL-60s. ‡ and ^†^ *P* < 0.05 with respect to all treatments except that bars marked by ‡ and † are not different from each other. ^#^*P* < 0.05 with respect to all treatments.

Studies evaluating the biosynthesis of sLe^X^ used two mAbs that recognize overlapping epitopes, mAbs HECA-452 (Fig. 4a) and CSLEX-1 (Fig. 4b). Here, both mAbs bound the GSLs prominently since the [G]^−^ cells displayed 60–75% loss of mAb binding while the [ON]^−^ cells that retain glycolipids bound the mAbs at 65–70% of WT HL-60 levels. The sialyl Lewis-X epitope is also likely expressed on N-glycans as mAb binding to [N]^−^ cells was reduced by 35–45%, with the N-glycan retaining [OG]^−^ cells binding both reagents at ~40% of WT levels. The O-glycans expressed a relatively small amount of the sLe^X^ epitope since the 25% reduction in HECA-452 and CSLEX-1 binding to [O]^−^ did not reach statistical significance. The binding of these mAbs to the O-glycan retaining [NG]^−^ cells was also only marginally higher than the TKOs which did not express sLe^X^. Overall, sLe^X^ expression on HL-60s likely follows the sequence: GSL (~60%) > N-glycans (~35%) > O-glycans (~5%).

Studies that assayed Le^X^ epitope expression on HL-60s suggest that this epitope is expressed primarily on the GSLs and N-glycans. Further, it is masked by the presence of O-linked carbohydrates. In this regard, consistent with the notion that the O-glycans mask the Le^X^ epitope, all single and double KO cells that lacked the O-glycans ([O]^−^, [OG]^−^, [ON]^−^) displayed 1.8–3.5 fold higher binding of the anti-Le^X^ mAb HI98 compared to WT. The N-glycans also displayed a smaller masking effect since the [N]^−^ cells bound the mAb at ~50% higher levels compared to WT. Further, mAb HI98 bound [ON]^−^ cells at high levels compared to the [O]^−^ cells. Finally, the Le^X^ epitope is likely expressed on both the GSLs and N-glycans since cells retaining the GSLs ([ON]^−^) and N-glycans ([OG]^−^) alone prominently bound the anti-Le^X^ mAb, whereas cells retaining O-glycans ([NG]^−^) and the triple-KO cell lines did not bind this reagent.

### P- and L-selectin mediated rolling is supported by O-linked glycans

**Figure 5:**
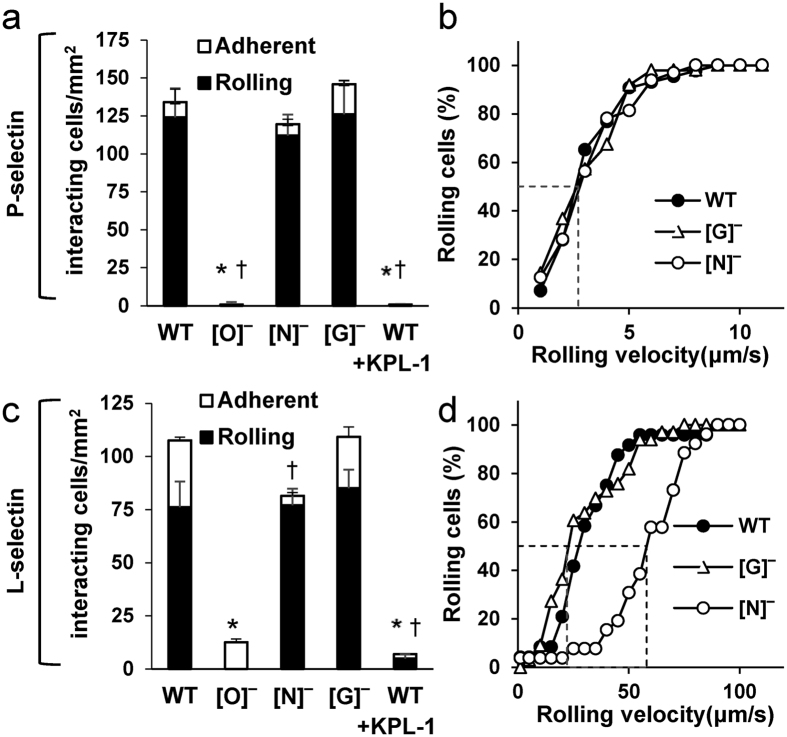
GlycoT KO rolling on recombinant P- and L-selectin. WT and KO HL-60s were perfused over (**a**,**b**) recombinant P-selectin or (**c**,**d**) L-selectin. Cell adhesion data are presented in left panels while cumulative rolling velocity plots appear on the right. Knocking out O-glycans (i.e. [O]^−^ cells) resulted in almost no interaction with either selectin. Knocking out N-glycans ([N]^−^ cells) augmented rolling velocity on L-selectin. *P < 0.05 with respect to all other cell types for rolling interactions. ^†^*P* < 0.05 with respect to all other cell types for adherent cells. Dashed lines in panels b and d are used to indicate the median rolling velocity.

The rolling phenotype of the HL-60 KOs on substrates bearing recombinant P- (Fig. 5a,b) and L-selectin (Fig. 5c,d) was evaluated using a microfluidics-based cell adhesion assay. Here, the absence of O-glycans ([O]^−^ cells) ablated cell binding to P-selectin (Fig. 5a). [N]^−^ and [G]^−^ cell rolling phenotype mirrored the WT-HL-60s (Fig. 5b).

The glycoconjugates contributing to L-selectin interactions were similar to that of P-selectin, with O-glycan deletion alone resulting in 85% reduction in interacting cells (Fig. 5b). Additionally, N-glycan deletion resulted in increased median rolling velocity on L-selectin, indicating a significant role for N-glycans in controlling cell binding. The GSLs did not influence cell adhesion to either L- or P-selectin under shear.

### Complementary roles for N-glycans, O-glycans and GSLs during E-selectin mediated rolling

**Figure 6:**
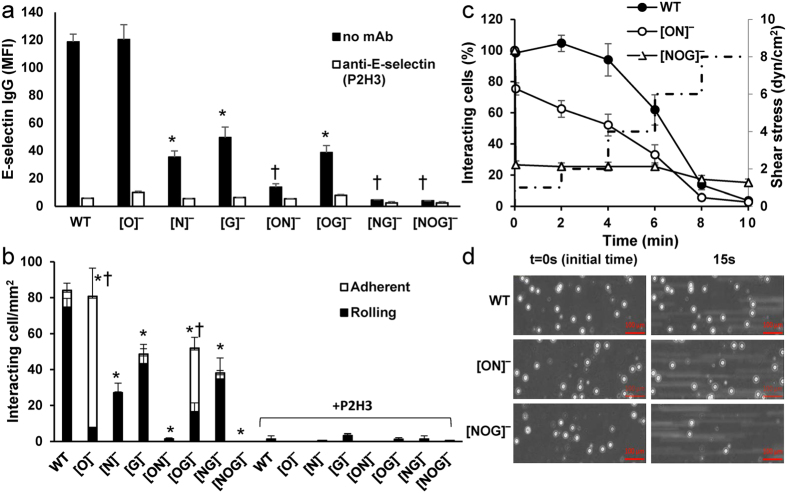
Cell adhesion to recombinant E-selectin-IgG under static and fluid shear conditions. (**a**) Human E-selectin-IgG binding to WT and KO cells measured using flow cytometry (Mean Fluorescence Intensity). P2H3 is an anti-E-selectin blocking mAb. * and ^†^ *P* < 0.05 with respect to all other treatments except bars indicated by these symbols are not different from each other. Error bars are too small to be visible in some cases. (**b**) WT and KO cells interacting with immobilized E-selectin-IgG in microfluidic flow cells at a wall shear stress of 1 dyn/cm^2^. Cells containing truncated N- and O-glycans (i.e. [ON]^−^ cells) were not captured from flow. * and ^†^ *P* < 0.05 for rolling and adherent cells respectively, with respect to WT cells. Statistics are not presented for anti-E-selectin blocking studies since cell adhesion was negligible in all cases. (**c**) Different cell types were captured under no-flow/static conditions for 2 min. prior to ramp-increase in wall shear stress starting with 1 dyn/cm^2^ between 0–2 min. Detailed shear protocol is shown on right axis using dashed line. (**d**) Representative figure from experiment in panel c at 0 and 15 s. [NOG]^−^ cells were released immediately upon initiation of flow. [ON]^−^and WT cells continued to roll for longer times.

Studies performed under static and hydrodynamic shear conditions evaluated the relative roles of different glycoconjugates to E-selectin mediated cell adhesion (Fig. 6).

In the static assay, flow cytometry measured the binding of recombinant E-Ig (E-selectin-human IgG_1_ fusion protein) to WT and mutant HL-60s in solution (Fig. 6a). Here, consistent with the sLe^X^ expression data, truncation of N-glycans ([N]^−^ cells) and glycolipids ([G]^−^) resulted in >50% decrease in E-Ig binding with O-glycan deletion ([O]^−^ cells) having no effect. When expressed alone, cells bearing N-glycans ([OG]^−^ cells) and glycolipids ([ON]^−^ cells) alone bound some amount of E-Ig, while cells with O-glycans ([NG]^−^) were non-binders. The molecular interaction in all cases was carbohydrate and selectin specific, since E-Ig binding was abolished by anti-E-selectin mAb P2H3, and the TKOs lacking glycans did not bind E-Ig.

In flow chamber studies, the HL-60s were captured under continuous flow onto E-Ig substrates at 1 dyn/cm^2^ (Fig. 6b). Cell adhesion was E-selectin dependent in all cases, as it was abolished by mAb P2H3. Here, N-glycans contributed to both cell tethering and rolling since HL-60s lacking N-glycans ([N]^−^ cells) displayed 60% reduction in rolling cell density. Cells bearing N-glycans alone ([OG]^−^) also displayed considerable cellular interactions. Whereas the absence of O-linked glycans ([O]^−^) did not alter the total number of interacting cells, both the [O]^−^ and [OG]^−^ cells facilitated greater firm cell arrest. In the presence of O-glycans alone ([NG]^−^ cells) some rolling was observed though the rolling velocity was 2-times higher than WT cells. Thus, O-glycans likely function with N-glycans to facilitate initial recruitment and rapid cell rolling in the initial phase, with some O-glycans also possibly masking the access of other leukocyte E-selectin ligands when rolling on the recombinant protein substrate. This may explain the enhanced selectin-dependent firm arrest in the O-glycan deficient cells^26^. The absence of GSLs resulted in reduced cell recruitment and faster rolling velocity (Fig. 6b). Finally, the cells bearing GSLs alone ([ON]^−^ cells) were not captured directly from flow (Fig. 6b), though these cells could sustain cell rolling up to 4–6 dyn/cm^2^ if captured under static conditions prior to the initiation of flow (Fig. 6c). In this stop-flow assay, the [NOG]^−^ cells, unlike the WT and [ON]^−^ cells, were immediately released upon shear initiation (Fig. 6c,d). Together, the data suggest that the GSLs likely contribute to stabilizing cell rolling but not the initial recruitment step.

### Glycans regulating cell adhesion to endothelial cells

**Figure 7:**
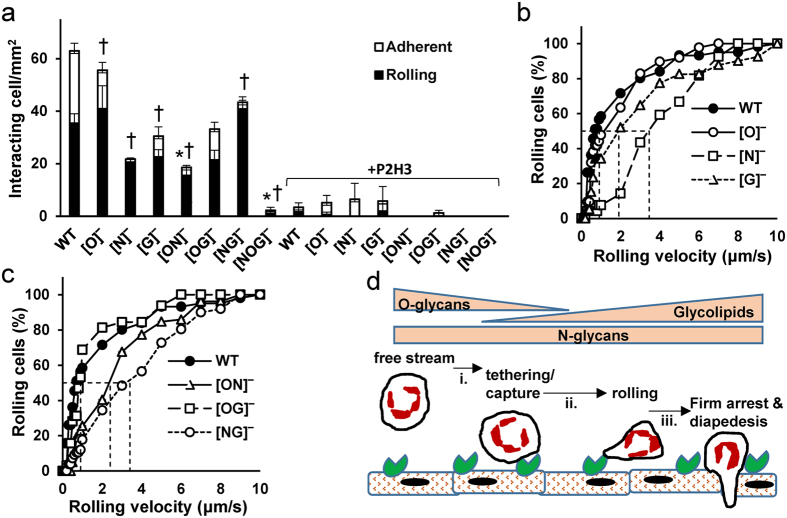
GlycoT KO cell rolling on E-selectin bearing stimulated HUVEC monolayers. WT and KO HL-60s were perfused over IL-1β stimulated HUVECs at 1 dyn/cm^2^. Both the density of rolling and adherent cells (panel a) and cell rolling velocity (panel b,c) were quantified. *P < 0.05 with respect to WT HL-60s for rolling cells. ^†^*P* < 0.05 with respect to WT HL-60s for adherent cells. Statistical analysis results are not presented for anti-E-selectin blocking studies since cell adhesion is low in all cases. (**d**) Model for the contribution of different E-selectin ligands in the multistep leukocyte-endothelial cell adhesion cascade. Here, O- and N-glycans contribute to tethering. N-glycans regulate cell rolling velocity, with O-glycans potentially shielding some E-selectin binding. GSLs control slow rolling and the transition to firm arrest. The model does not account for biochemical processes regulating leukocyte activation.

The final assay characterized WT and mutant HL-60 adhesion to IL-1β stimulated HUVECs, an E-selectin specific molecular interaction that is blocked by mAb P2H3 (Fig. 7a). In this physiological cell-based system, similar to the E-Ig flow chamber studies, the deletion of the N-glycans ([N]^−^ cells) and GSLs ([G]^−^ cells) reduced the density of interacting cells. N-glycan and GSL deletion also increased leukocyte rolling velocity confirming contributions from these glycoconjugates to human myeloid cell rolling (Fig. 7b). O-glycan deletion ([O]^−^ cells) itself had a negligible effect, similar to the E-Ig assay. Among the dual KOs, all cells were able to recruit leukocytes from the flow stream with cells bearing N- ([OG]^−^) and O-glycans ([NG]^−^) alone being most efficacious. However, the [NG]^−^ cells displayed the fastest rolling velocity (Fig. 7c), indicating that the O-glycans are likely to be only important during the initial recruitment and rapid rolling phase. Additionally, unlike the E-Ig assay (Fig. 6b), the [ON]^−^ cells were also recruited onto endothelial cells presumably due to endothelial substrate roughness that enables enhanced interaction with GSLs on the granulocyte cell-surface. Finally, similar to the E-Ig assay, the N-glycans were the major regulator of cell rolling velocity as cells bearing N-glycans ([OG]^−^) displayed rolling velocities comparable to WT HL-60s\, unlike the O-glycan ([NG]^−^) and GSL ([ON]^−^) bearing cells which rolled 3–4 fold faster (Fig. 7c). The absence of N-glycans in [N]^−^, [ON]^−^ and [NG]^−^ also prevented HL-60 firm arrest (Fig. 7a), suggesting a key role for such glycoconjugates during myeloid cell transition to firm arrest. A model for myeloid cell recruitment and adhesion on E-selectin is presented in Fig. 7d based on studies performed using E-Ig and HUVECs.

## Discussion

The study introduces a CRISPR-Cas9 toolkit that may be useful in dissecting the relative contributions of N-glycans, O-glycans and GSLs in a variety of cellular processes. In particular, it defines specific gene targets in these different pathways, the abrogation of which does not compromise cell survival or growth potential. In this regard, even the triple knockout cells lacking extended O-, N- and lipid-linked glycans ([NOG]^−^, [GON]^−^) were viable. Thus, terminal glycan structures may not be necessary for cell growth and proliferation during *in vitro* culture. A broader range of studies is necessary to confirm this finding in additional cell types. To account for the possibility that the deletion of a particular glycan family can result in compensation by other families of glycoconjugates, the study generated both loss-of-function cell types that lacked single glycan types ([O]^−^, [N]^−^ and [G]^−^ cells), and corresponding retention-of-function cells that specifically contained either only O-linked ([NG]^−^), N-linked ([OG]^−^) or GSL ([ON]^−^) structures. Overall, the advantage of the CRISPR-Cas9 method is that it allows efficient genetic manipulation of human cells, and results in the complete deletion of selected enzyme activities unlike previous technologies, such as shRNA or chemical inhibitors, which only resulted in partial attenuation of function^6^,^8^,^10^,^11^.

Using human myeloid cell binding to selectins as an example, the study confirms that the O-linked core-2 sLe^X^ glycan located at the N-terminus of PSGL-1 carries the prominent L- and P-selectin ligand on human leukocytes. This is consistent with prior literature^11^,^27^,^28^. This glycan likely facilitates the direct capture of myeloid leukocytes to P-selectin at sites of vascular inflammation and it additionally amplifies leukocyte recruitment through L-selectin mediated secondary leukocyte-leukocyte capture^29^,^30^. In addition, this study suggests that a yet unidentified N-linked glycan also facilitates secondary cell capture.

With regard to E-selectin, the study highlights distinct roles for O-glycans, N-glycans and GSLs in the different phases of the leukocyte adhesion cascade (Fig. 7d). Here, the O- and N-glycans were the major players facilitating leukocyte recruitment since cells bearing either glycoconjugate alone ([NG]^−^ or [OG]^−^ cells) could readily capture leukocytes onto both E-selectin and HUVEC substrates. Among these, the contribution of N-glycans to recruitment was more prominent since the [N]^−^ mutants displayed more severe reduction in the interacting cell density compared to the [O]^−^ cells. Depletion of O-glycans in the [O]^−^ and [OG]^−^ cells also resulted in greater firm arrest on E-Ig but not HUVEC monolayers, suggesting some differences in the nature of leukocyte binding to these two substrates. These differences may be due to different conformations or presentations of E-selectin on these substrates^31^, or the presence of additional epitopes (like the Fc portion of E-Ig) that engage HL-60s when the O-glycans are absent. Finally, to rule out the possibility that the truncation of N-glycans and GSLs results in altered O-glycan structures that contribute to the model in Fig. 7d, mass spectrometry was undertaken using the recently developed CORA method (cellular O-glycoproteome reporter assay^32^, Supplemental Fig. S4). Here, as expected, O-glycans were absent in all cells that lacked COSMC. All other KO cell types displayed O-glycans nearly identical to WT HL-60.

As opposed to the glycoproteins, the GSLs were more important during the slow rolling rather than the initial cell recruitment step. In support of this, the glycolipid expressing [ON]^−^ cells could not be captured directly from flow onto E-Ig substrates. However, these cells once captured under static conditions, sustained robust rolling on E-selectin up to 4–6 dyn/cm^2^. Such slow rolling may enhance leukocyte-endothelium contact, facilitating cell activation and the conversion of rolling cells to firm arrest. In addition to the GSLs, the N-glycans also controlled cell rolling since all cell types lacking the N-glycans ([N]^−^ [NG]^−^, [ON]^−^) displayed faster rolling on E-selectin bearing HUVECs compared to wild-type HL-60s. All cell adhesion under flow was carbohydrate dependent as the [NOG]^−^ cells did not bind selectins in static or shear based assays.

The truncation of specific glycan families on leukocytes may reveal the presence of cryptic, underlying structures that were previously hidden. This was most prominently noted using the anti-Le^X^ mAb HI98 which recognizes carbohydrate epitopes on N-glycans and GSLs (Fig. 4c). Here, all cells lacking extended O-glycans displayed a 200–350% increase in anti-Le^X^ binding. The O-glycans themselves did not prominently express the Le^X^ epitope since the [NG]^−^ dual KOs did not bind HI98. Unlike Le^X^, the binding of neither the anti-sLe^X^ mAbs (HECA-452 or CSLEX-1) nor E-IgG was increased above WT levels upon knocking out any of the glycosyltransferases. Thus, these E-selectin-binding epitopes are not likely to be cryptic in leukocytes. Finally, consistent with previous mass spectrometry data^10^, sLe^X^ was expressed on all types of leukocyte glycoconjugates on HL-60s especially the GSLs and N-glycans with lower expression on O-glycans.

While the current study shows that different families of glycoconjugates regulate distinct phases of the E-selectin dependent cell adhesion cascade, it does not focus on the exact leukocyte macromolecules recognized by E-selectin^19^,^33^. In this regard, previous studies show that PSGL-1, E-selectin Ligand-1 (ESL-1), and CD44 are the major E-selectin ligands in mice^34^. The corresponding ligands in humans are yet unknown^14^,^19^, since PSGL-1 is only a minor ligand for human E-selectin^11^, ESL-1 has no homolog in humans^35^ and human CD44 is important for hematopoietic stem cells but not mature granulocyte rolling^13^. Additionally, whereas murine myeloid cell rolling on E-selectin is abolished by pronase, human leukocytes roll robustly on E-selectin after similar protease treatment^18^. Thus, the GSLs may regulate some aspects of cell rolling, particularly the slow rolling. Currently, N- and O-glycans that engage human E-selectin under flow are thought to exist on leukocyte CD43, integrin Mac-1 and/or L-selectin^14^,^19^,^33^, though this awaits confirmation using gene knockout approaches. Additionally, while the current study is limited to HL-60 cells, newer methods have to be developed so that the CRISPR-Cas technology may be applied to simultaneously edit multiple genes in short-lived primary human neutrophils and track these mutants. Overall, the identification of the glycoconjugates regulating leukocyte adhesion can lead to new ways of targeting inflammation using metabolic^7^,^36^ and competitive inhibitors^37^,^38^.

## Methods

### Cell culture

HL60 cells (ATCC, Manassas, VA) were cultured in Iscove’s Modified Dulbecco’s Medium (IMDM) with 10% FBS. Human Umbilical Vein Endothelial Cells (HUVEC) were maintained in EBM-2 media (Lonza, Walkersville, MD).

### Flow Cytometry

Mouse monoclonal antibodies (mAbs) used in this study include FITC-conjugated anti-Lewis-X (Le^X^)/CD15 mAb HI98, unconjugated anti-sialyl Lewis X (sLe^X^)/CD15s mAb CSLEX-1, FITC conjugated rat anti-Cutaneous Lymphocyte Antigen (CLA) mAb HECA-452, anti-CD62L/L-selectin clone DREG-56-FITC, anti-CD44 mAb G44-26-FITC, anti-CD43 mAb MEM59-FITC (Biolegend, San Diego, CA), anti-CD11b mAb D11-PE (phycoerythrin), anti-CD162 clone KPL-1-PE and isotype-matched controls. All mAbs were from BD Biosciences (San Diego, CA), unless noted otherwise. FITC-conjugated *Vicia Villosa* lectin (VVA, binds GalNAcα1-Ser/Thr), *Phaseolus vulgaris* leucoagglutinin (L-PHA, binds Galβ4GlcNAcβ6 (GlcNAcβ2Manα3) Manα3), *Erythrina cristagalli* lectin (ECL, binds Galβ4GlcNAc), *Peanut Agglutinin* (PNA, binds Galβ1,3GalNAc), Concanavalin A (ConA, binds αMan and αGlc), and biotinylated *Maackia amurensis* lectin II (MALII, binds α2,3-linked sialic acid) were from Vector labs (Burlingame, CA)^39^. E-selectin-IgG (E-Ig) was purchased from R&D Systems (Minneapolis, MN). For flow cytometry, cells were incubated with 5–25 μg/ml fluorescently labeled mAbs or lectins for 20 min. in HEPES buffer (30 mM HEPES, 10 mM glucose, 110 mM NaCl, 10 mM KCl, 1 mM MgCl_2_, pH 7.2) containing 1.5 mM CaCl_2_ and 0.1% human serum albumin (HSA) at 4 °C for 20 min. prior to analysis. Studies with unlabeled mAbs or biotinylated lectins involved a second 20 min. incubation step with 1:250 diluted Alexa-488 conjugated anti-mouse secondary antibody (Invitrogen, Carlsbad, CA) or 10 μg/ml fluorescein conjugated goat anti-biotin antibody (Vector Labs). For the selectin binding measurements, 3 μg/ml E-Ig was incubated with 10 μg/ml goat anti-human PerCP Ab (Jackson ImmunoResearch, West Grove, PA) for 10 min at room temperature in HEPES buffer containing 1.5 mM CaCl_2_ and 1% goat serum^40^. 10^6^ WT or KO cells/ml was then added to this suspension for 10 min at RT (room temperature) prior to cytometry analysis. 5 μg/ml mouse anti-human CD62E blocking mAb P2H3 (eBioscience, San Diego, CA) was added to confirm binding specificity.

### Genomic deletion of glycoTs in leukocytes

Target sites on human *COSMC*, *UGCG* and *MGAT1* were determined using the CRISPR Design tool by minimizing exonic off-target effects^41^. Target sequences were cloned into the pX330-U6-Chimeric_BB-CBh-hSpCas9 vector (Addgene, Plasmid# 42230) after BbsI digestion. COSMC-KO ([O]^−^cells), MGAT1-KO ([N]^−^cells) and UGCG-KO ([G]^−^cells) single KO cell lines were created by electroporating WT HL-60s with vectors containing target sequences (Fig. 2) against the respective genes. COSMC/UGCG-KO dual KOs (i.e. ([OG]^−^cells) were made by co-transfection of HL-60 with an equimolar plasmid mixture containing *COSMC* and *UGCG* targeting plasmids. Dual KO COSMC/MGAT1-KO ([ON]^−^) and MGAT1/UGCG-KO ([NG]^−^), and triple KO MGAT1/COSMC/UGCG-KO ([NOG]^−^) and UGCG/COSMC/MGAT1-KO ([GON]^−^) cell lines were produced by transfecting [N]^−^, [G]^−^, [OG]^−^ or [ON]^−^ cells, respectively, with a vector targeting either *COSMC*, *MGAT1* or *UGCG*. In all cases, HL60 growth media was changed to RPMI-1640 with 10% FBS at least 48 h prior to electroporation using the Neon Transfection System (Invitrogen). Cells were electroporated according to the manufacturer’s recommendation. After electroporation, cells were cultured in RPMI 1640 with 10% FBS without antibiotics overnight and then supplemented with 50/50 (v/v) mixture of fresh and HL-60 cell-culture conditioned RPMI 1640 (conditioned media). Approximately 2–3 weeks thereafter, both single-cell and bulk-cell FACS sorting (BD FACSAria) was performed using high VVA-FITC binding to select for the COSMC-KOs and low L-PHA-FITC staining to detect Mgat1 deletion. Conditioned media was necessary for single cell cultures to maintain cell viability. After scale up, culture media was changed back to IMDM with 10% FBS for all functional studies. To characterize the KOs, the region surrounding the CRISPR target site was PCR amplified from genomic DNA with primers in Supplemental Table S1 and sequenced. Clones were checked for exonic off-target mutations predicted by the CRISPR Design tool. Off-target sites and PCR primers for *COSMC* and *UGCG* gRNA are listed in Supplementary Tables S2 and S3, respectively. *MGAT1* gRNA lacked any predicted exonic off-targets. Off-target editing was absent in all clones characterized in this manuscript (Supp. Tables S2, S3).

### C1GalT1, Mgat1 and UGCG Enzymatic Assays

~50 × 10^6^ WT and KO HL-60s were lysed in 100 mM Tris-maleate buffer (pH 7.2) containing 2% Triton-X 100 and 1 mM PMSF using a dounce homogenizer. Homogenates were centrifuged at 14,000 × g for 30 min at 4 °C. Total protein in the supernatants was measured and adjusted to 10 mg/ml total protein with lysis buffer. Lysates were snap frozen in liquid nitrogen and stored at −80 °C.

The C1GalT1 and Mgat1 enzyme assays were radioactivity-based similar to prior work^20^,^42^. Briefly, 1.5 μl of lysate was incubated with either 0.4 mM Benzyl-α-GalNAc and 0.2 μCi ^14^C-UDP-Gal for the C1GalT1 assay, or 0.5 mM Mannose-3-octyl and 0.2 μCi ^14^C-UDP-GlcNAc for Mgat1 activity, in 0.1 M 2-ethanesulfonic acid (MES) buffer (pH 6.8) containing 2 mM ATP and 20 mM MnCl_2_ for 4 h at RT. Reactions containing no lysate or no acceptor served as negative controls. At the end-point, the reaction mixtures were spotted on silica gel 60 high performance thin layer chromatography plates (HPTLC, EMD Chemicals, Gibbstown, NJ), resolved in CHCl_3_:CH_3_OH:H_2_O (5:4:1) and visualized by storage phosphor.

The UGCG enzymology assays used fluorescent C6-NBD-ceramide substrate (Avanti Polar Lipids, Alabaster, Alabama)^43^. Here, 12.5 μg C6-NBD Ceramide and 125 μg lecithin (Avanti Polar Lipids) were dried down and then dissolved in 25 μL ethanol followed by addition of 225 μl water. Liposomes were then formed by sonication for 4 h at 4 °C. A 100 μL reaction mixture containing 20 μL liposome, 5 μl cell lysate and 500 μM UDP-Glucose in 20 mM Tris-HCl (pH 7.5) buffer was incubated overnight at 30 °C. The reaction was stopped by addition of 100 μL CHCl_3_:CH_3_OH (2:1) and phase separated by centrifugation at 14000 × g for 10 min. The bottom organic layer was collected, solvent evaporated and resuspended in 4 μl CHCl_3_. The mixture was resolved on a silica gel 60 plate with CHCl_3_:CH_3_OH:12 mM MgCl_2_ in H_2_O (65:25:4). TLC plate images were imaged using a UVP ChemiDoc imaging system (Upland, CA) with 302 nm excitation and 570–640 nm emission. C6-NBD-GlcCer product standard (Avanti Polar Lipids) served as the positive control. The negative control lacked cell lysate.

### Microfluidic flow chamber studies

WT and variant HL-60 cells were perfused over substrates bearing physiological levels of P-, L- and E-selectin, or IL-1β stimulated human umbilical vein endothelial cells (HUVECs) as described previously^11^. Briefly, P- and L-selectin substrates were created by adsorbing tissue culture plates with 25 μg/ml recombinant P- or 10 μg/ml L-selectin (R&D Systems) at RT for 4 h. E-selectin substrates were prepared by addition of 1.3 μg/ml goat anti-human Fc Ab to the plates at 4 °C overnight prior to capturing 2 μg/ml E-Ig (E-selectin IgG fusion protein) for 4 h at 4 °C. All substrates were blocked with HEPES buffer containing 1–3% BSA (bovine serum albumin) for 1 h at RT prior to experimentation. HUVECs were stimulated with 5 U/ml IL-1β for 4 h at 37 °C to upregulate E-selectin.

Cell adhesion studies utilized a custom microfluidics flow chamber (600 μm width × 100 μm height × 1 cm length) that was vacuum sealed onto the selectin-bearing substrate on the stage of an AxioObserver Z1 microscope^11^. A 10 μl pipette tip served as the inlet cell suspension reservoir. The outlet was connected to a 500 μl Hamilton syringe mounted on a Harvard Apparatus syringe pump (Pump 11, Holliston, MA). Following 10 min. equilibration using HEPES buffer containing 1.5 mM Ca^2+^ and 0.1% HSA, 2–5 × 10^6^ HL-60 s/ml were perfused at a wall shear stress of 1–2 dyn/cm^2^ in the same buffer. For blocking studies, the substrates were incubated with 10 μg/ml blocking mAbs P2H3 (anti-E-selectin), G1 (anti-P-selectin) or DREG-56 (anti-L-selectin) for 20 min prior to cell perfusion. All data were captured at 2 fps using a PCO edge sCMOS camera (PCO AG, Kelheim, Germany) and ZEN 2012 image acquisition software (Carl Zeiss). Data analysis was performed using ImageJ. Here, interacting cells were defined as cells traveling at velocities less than the theoretical free stream velocity of non-interacting 10 μm diameter spheres at the chamber wall^11^. ‘Adherent’ cells were defined to move less than one cell diameter in a 10 s period. The remaining cells were classified to be ‘rolling’. Rolling velocity was calculated by tracking individual cell trajectories.

### O-glycome profiling

HL-60 O-glycan structures were measured using the Cellular O-glycome Reporter (CORA) method described recently^32^. Here, 50–80 μM per-acetylated Benzyl-α-GalNAc was fed to HL-60 cells plated at an initial density of 0.2 × 10^6 ^cells/ml in Advanced DMEM media without phenol red or serum proteins for 3 days. Benzyl-α-GalNAc derivatives were then purified from 5 mL of cell culture supernatant using the Sep-Pak C18 method described previously^32^. Eluates obtained using 50% methanol were permethylated^32^. Products were analyzed using an LCQ ion trap mass spectrometer (Thermo). All product structures were confirmed using MS^n^ (n = 2-4) spectral analysis.

### Statistics

Data are presented as mean ± SEM for ≥3 experiments. One-way ANOVA followed by the Tukey post-test was applied for multiple comparisons. *P* < 0.05 was considered to be statistically significant.

## Additional Information

**How to cite this article**: Stolfa, G. *et al*. Using CRISPR-Cas9 to quantify the contributions of O-glycans, N-glycans and Glycosphingolipids to human leukocyte-endothelium adhesion. *Sci. Rep.* **6**, 30392; doi: 10.1038/srep30392 (2016).

## Supplementary Material

Supplementary Information
