# Supplementary material for: Using CRISPR-Cas9 to quantify the contributions of O-glycans, N-glycans and Glycosphingolipids to human leukocyte-endothelium adhesion
Source: Sci Rep. 2016 Jul 26;6:30392. doi: 10.1038/srep30392 (PMC4960646; doi:10.1038/srep30392)
Supplement: Supplementary Information [file srep30392-s1.pdf]

Using CRISPR-Cas9 to quantify the contributions of O-glycans, N-glycans and Glycosphingolipids to human leukocyte-endothelium adhesion

Gino Stolfa[*], Nandini Mondal[*], Yuqi Zhu, Xinheng Yu, Alexander Buffone, Jr. and Sriram Neelamegham

* Joint first authors

Department of Chemical & Biological Engineering, and Clinical & Translational Research Center, State University of New York, Buffalo, NY 14260, USA

Table S1. Target Genomic DNA PCR Primers

Gene	Forward Primer	Reverse Primer
MGAT1	TTCCTCCACCCTCACTCTTG	TAATGCAGCAGCTTGTCCAG
COSMC	AAACAAACTTCTCCATAGAGG	TGCTTTGTCACAGTGTTTGG
UGCG	AGCCGGGAGACCGCAGCAC	CAGGAATTCTGTCCTGCTCACCACC

Supplementary Table S2. COSMC Exonic Off-Target sequence verification

Gene	Target Site	Forward Primer	Reverse Primer	[O]$^{-}$	[ON]$^{-}$	[OG]$^{-}$	[NOG]$^{-}$	[GON]$^{-}$
RC3H1	CAATCTTTGGGCAGCAGTAAGGG	TGGGTCTGAACAGCACTACTC	GGAATTGCCCAAGCCATGAAC	NE	NE	NE	NE	NE
MSN	GGTTCTTTGGTCTGCAGTACCAG	AAGGAGGAGGTGGCAGAAATC	CCTCGGTCCTTATTCTGATCC	NE	NE	NE	NE	NE
CSF1R	TAGTTGTTGGGCTGCAGCAAGGG	CTCCCACCCTCAGGACTATAC	CCTCACCTTCCCAAGTTTCAG	NE	NE	NE	NE	NE
C15orf52	GGGTCTTTGTTCTGCAGGAAAAG	GGGACAGAGACCCATTGTTTG	GCTCACCTCTGCTTTGTTCTC	NE	NE	NE	NE	NE

Each potential off-target gene (listed in first column) was PCR amplified using indicated forward and reverse primers. This was performed for all cell lines listed in the last five columns. Sequencing was performed to identify any off-target editing. NE = no off-target editing was detected in any case.

Supplementary Table S3. UGCG Exonic Off-Targets sequence verification

Gene	Target Site	Fwd Primer	Rvs Primer	[G]$^{-}$	[NG]$^{-}$	[OG]$^{-}$	[NOG]$^{-}$	[GON]$^{-}$
PRKDC	CCAGTGGCTGATGCATATCAGAG	TAAGTACCCTTCTGCCCTCAC	ACAGCAAGTGCACCTGTGTAG	NE	NE	NE	NE	NE
PLXNA3	ACTGTGGCTGGTGCATCTCAGAG	CTTCCCTCCTAAGGCAATTGG	ACAAGAGGCCCAGGTTGTCAC	NE	NE	NE	NE	NE
HOXA2	CCTGTCGCTGATACATTTCAAAG	CCCATACGGCTGTAATCAGTG	CCGCCTTCTTCTCCTTCATCC	NE	NE	NE	NE	NE
KIF17	GCCGGGGCTGGGGCATTTCAGGG	GACATAGTTGCTGGGTCTGAG	GGGAACAGGGAAGGGAATGTG	NE	NE	NE	NE	NE
BTBD11	GCTGCAGGTGATGCATTGCACGG	GTGTTGGCCACAACATAGAGG	GTGGGCAGTGCATGAAATAGC	NE	NE	NE	NE	NE

PCR amplification of off-target sites was performed as in Table S2. NE = no off-target editing was detected in any case.

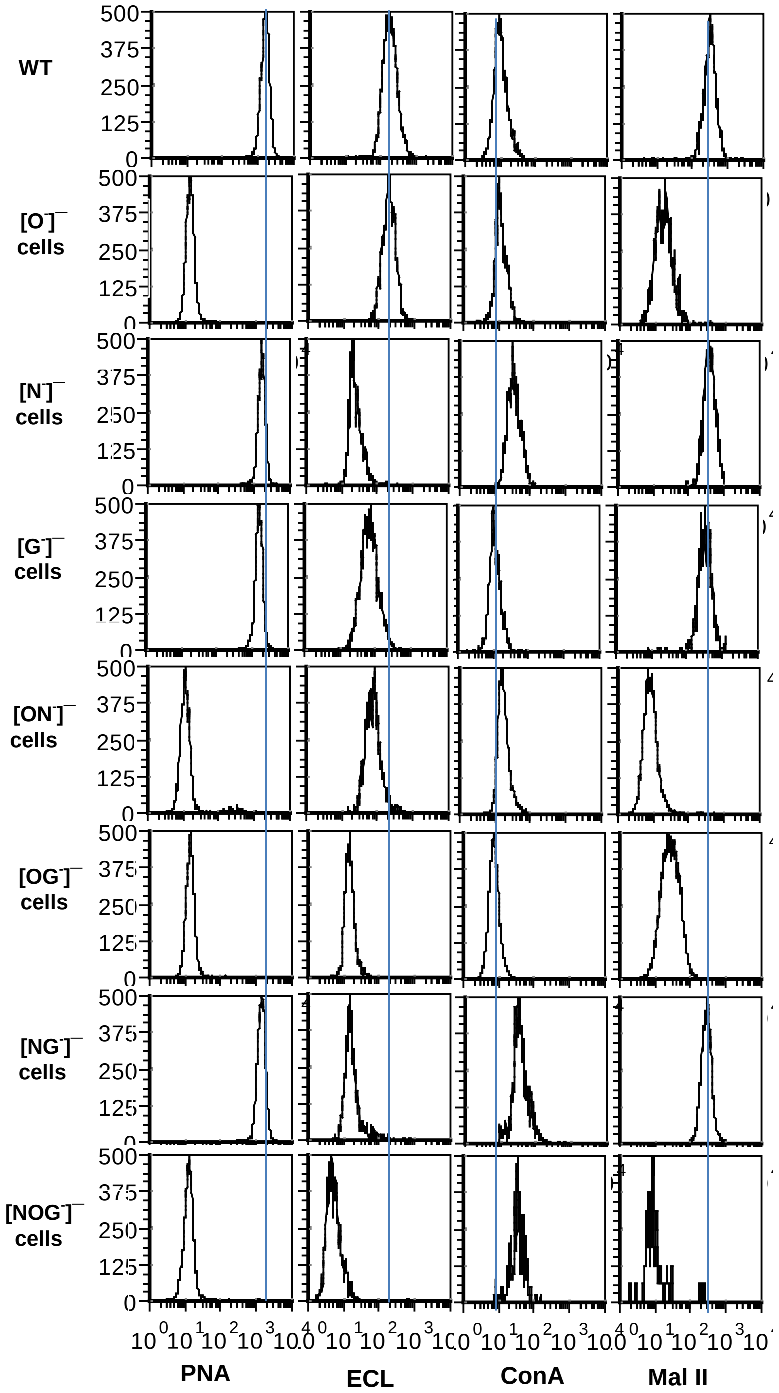

***Supplemental Figure S1. Lectin staining of KO cells measured using flow cytometry*:** PNA binds Galβ1,3GalNAc on core-1 O-glycans. It is detected on cell surface after de-sialylation using α2,3/6/8/9 *Arthrobacter ureafaciens*. ECL recognizes unsialylated lactosamine/Galβ1,4GlcNAc. ConA binds high mannose N-linked glycans. Mal-II stains α-2,3 linked sialylated glycans. PNA and Mal-II binding are reduced in the O-glycan KOs. ConA staining is augmented in the KOs containing truncated N-glycans. ECL staining is diminished in cells bearing truncated N-glycans and also glycolipids.

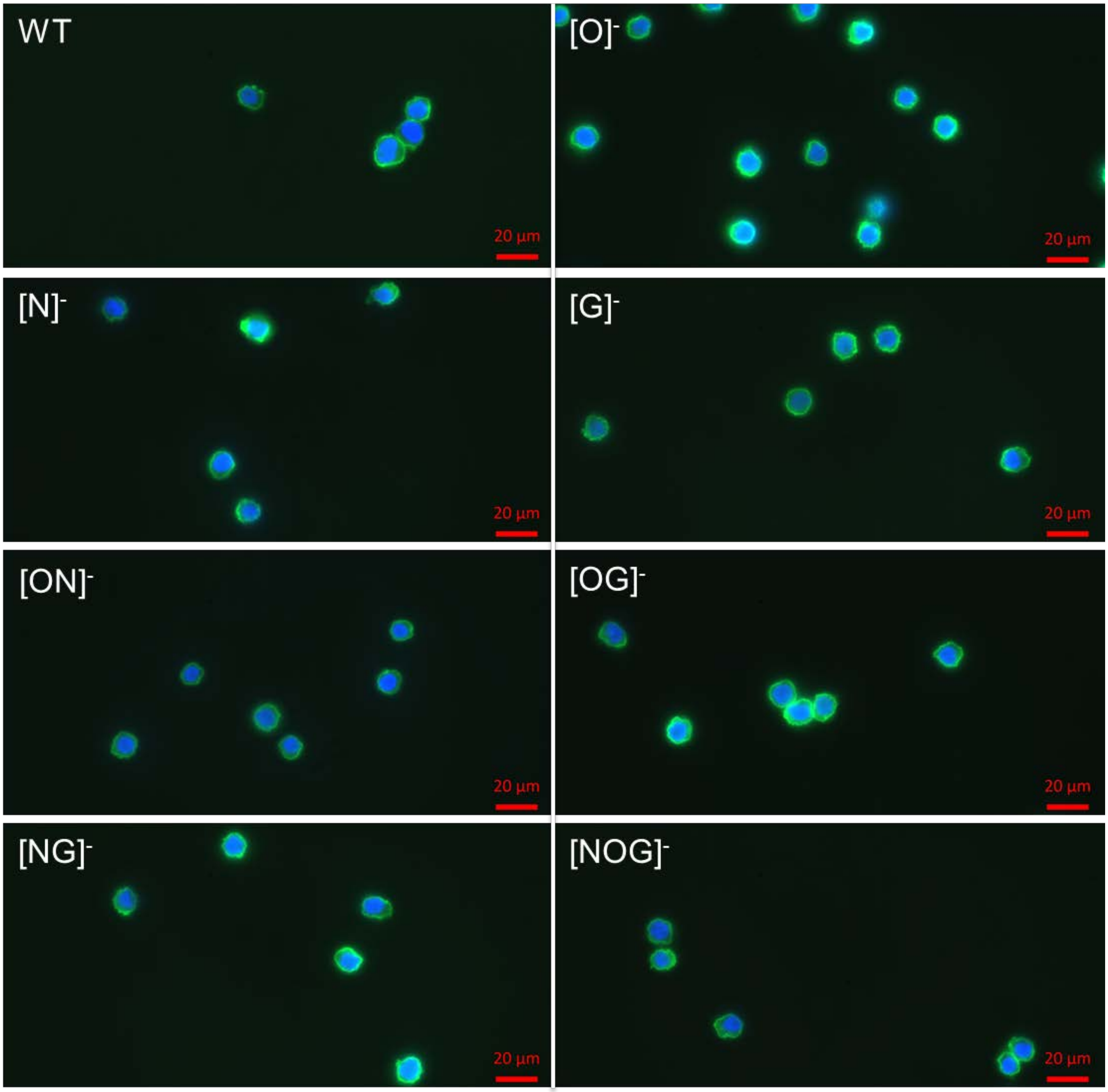

***Supplemental Figure S2. Micrographs of all KO cells*:** Blue is DAPI. Phalloidin-Alexa 488 staining is shown in green. All images were acquired using a 66X/1.4NA objective. Cell size in all cases was ~10µm (measured using a cell counter) and there was no remarkable difference among the cell lines in terms of growth or viability.

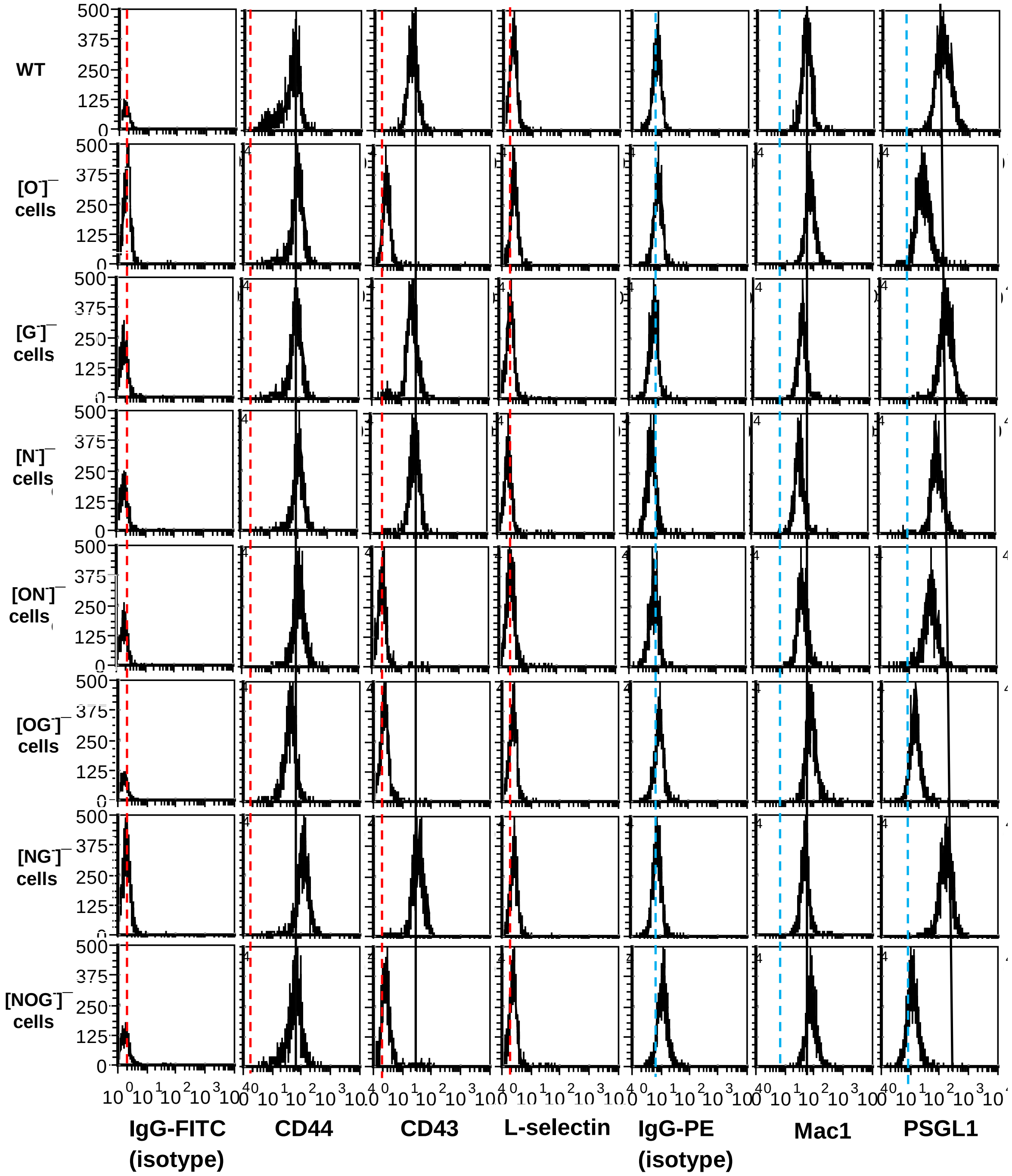

Supplemental Figure S3. Cell surface expression of potential E-selectin glycoprotein ligands **measured using flow cytometry:** Dotted lines mark the background fluorescence of isotype control Abs: Vertical solid lines mark the fluorescence of wild-type cells for the different mAbs. FITC conjugates were used in the case of anti-CD44 (clone G44-26), CD43 (MEM59) and L-selectin (DREG-56), and PE-conjugate reagents were used in the case of the CD11b/Mac-1 (D11) and CD162/PSGL-1 (KPL-1) mAbs.

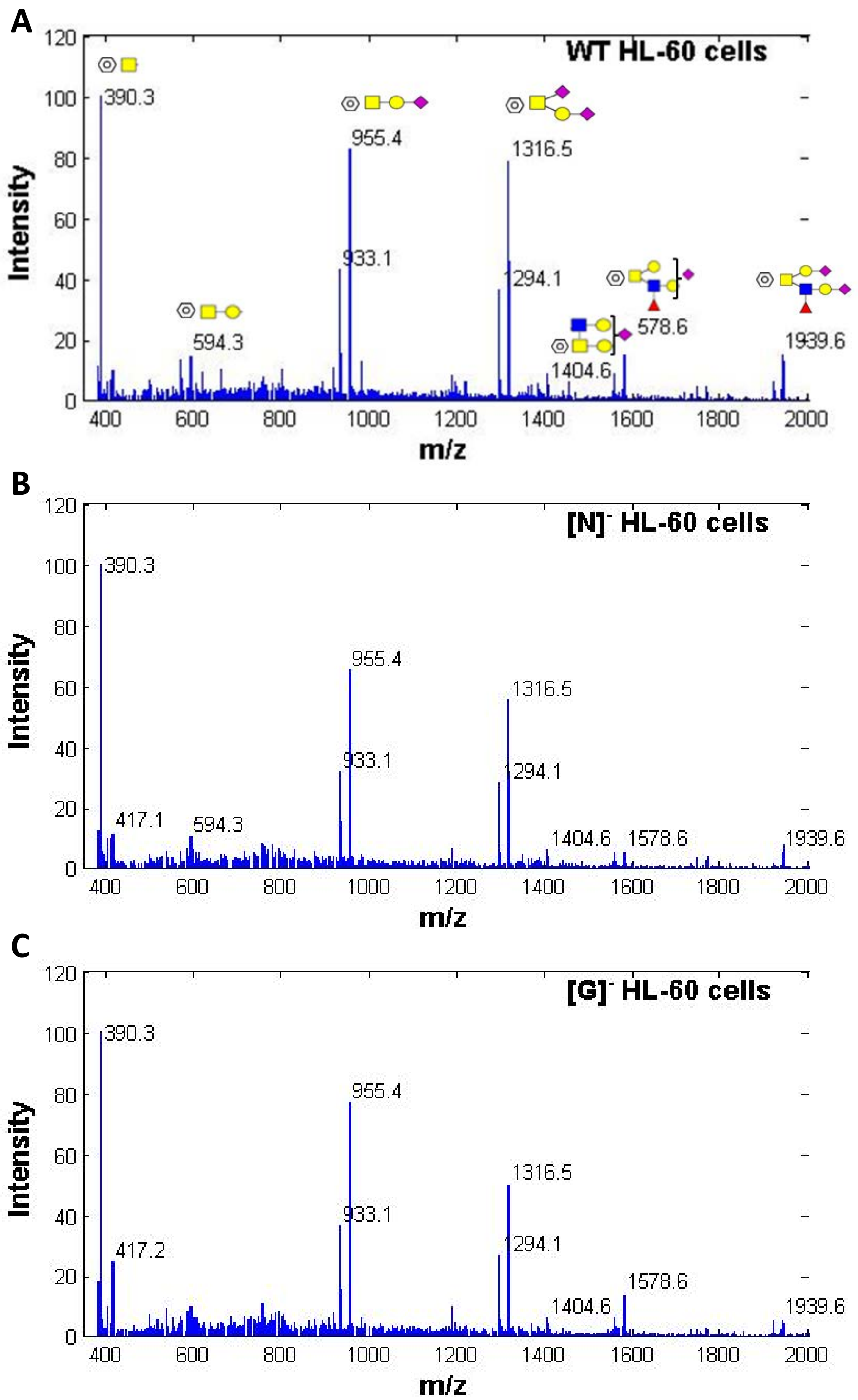

***Supplemental Figure S4. Cellular O-glycome Reporter Assay (CORA)*:** Benzyl-α-GalNAc fed to cells in culture for 3 days was purified from culture supernatant. Glycosylated products formed on this substrate were analyzed using mass spectrometry. Under the experimental conditions, the most prominent glycans were sodium adducts ($[M+Na]^+$), shown using the Consortium for Functional Glycomics (CFG) symbolic nomenclature. The corresponding $[M+H]^+$ ions appear 22Da below the sodium adduct. All glycan structures drawn in panel **A** were verified using MS^n analysis (n=2-4). These same structures were observed in panels B, C and E (cartoons not shown). When unambiguous determination was not possible, curly brackets are used to represent isomeric structures. CORA data are presented for WT HL-60s (panel A) and all knockouts (B-H). Whereas the O-glycan knockouts did not form glycosylated products on the Benzyl-α-GalNAc substrate, the O-glycan profile of all other cell types closely resembled the WT HL-60s.

D

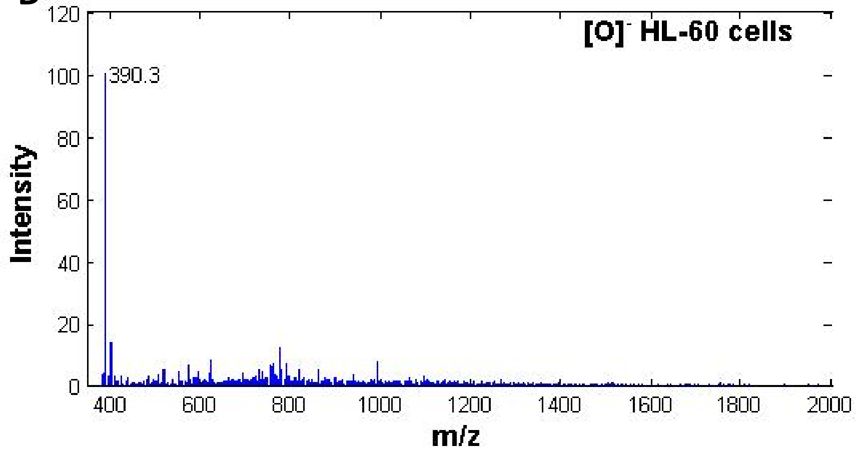

E

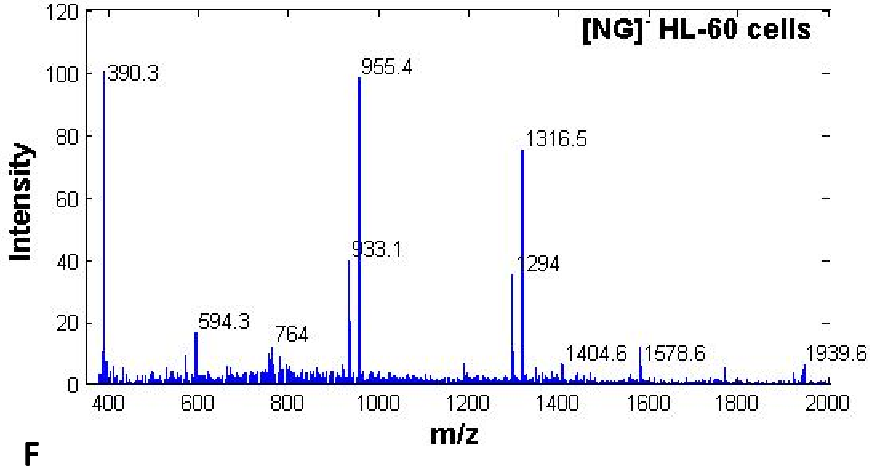

F

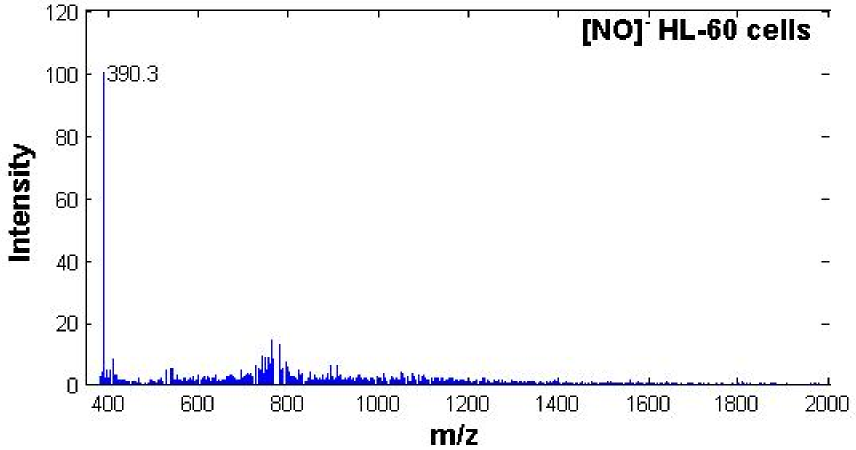

Supplemental Figure S4 (continued)

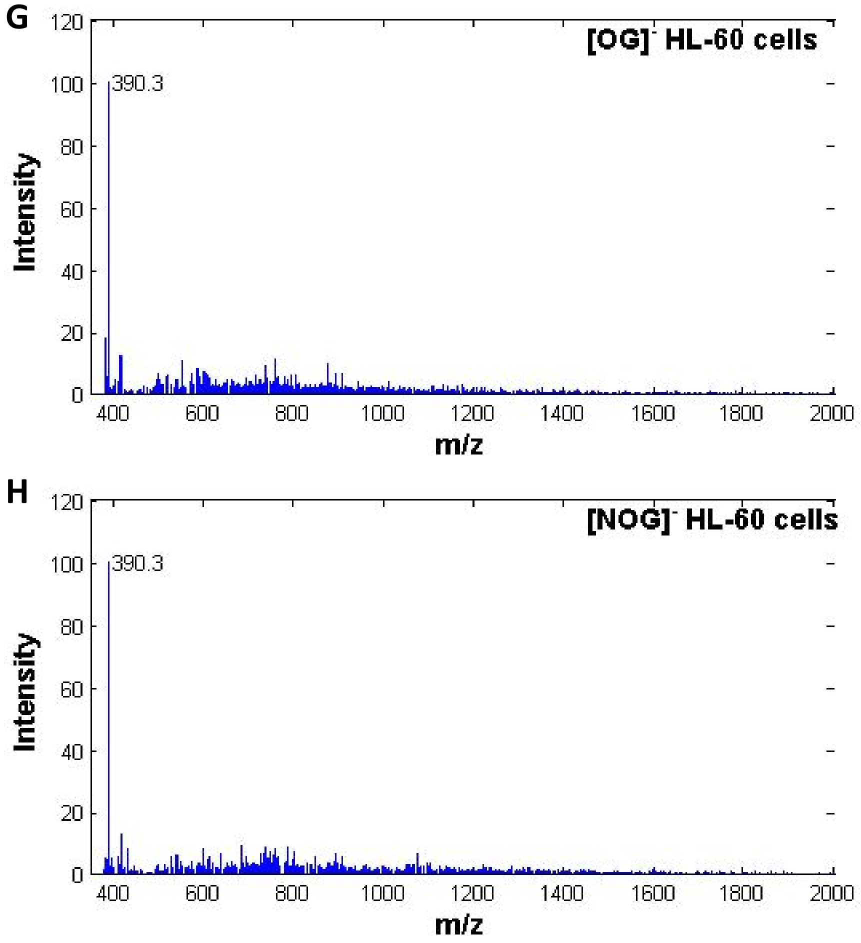

Supplemental Figure S4 (continued)
